# Determinants and dynamics of pancreatic islet architecture

**DOI:** 10.1080/19382014.2022.2030649

**Published:** 2022-03-08

**Authors:** Melissa T. Adams, Barak Blum

**Affiliations:** Department of Cell and Regenerative Biology, University of Wisconsin-Madison, Madison, WI, USA

**Keywords:** Islets of Langerhans, islet development, islet architecture, diabetes, islet morphogenesis

## Abstract

The islets of Langerhans are highly organized structures that have species-specific, three-dimensional tissue architecture. Islet architecture is critical for proper hormone secretion in response to nutritional stimuli. Islet architecture is disrupted in all types of diabetes mellitus and in cadaveric islets for transplantation during isolation, culture, and perfusion, limiting patient outcomes. Moreover, recapitulating native islet architecture remains a key challenge for *in vitro* generation of islets from stem cells. In this review, we discuss work that has led to the current understanding of determinants of pancreatic islet architecture, and how this architecture is maintained or disrupted during tissue remodeling in response to normal and pathological metabolic changes. We further discuss both empirical and modeling data that highlight the importance of islet architecture for islet function.

## Introduction

Glucose homeostasis in vertebrates is made possible by the islets of Langerhans. These small clusters of endocrine cells reside in the pancreas, surrounded by exocrine tissue. In contrast to the exocrine tissue, which secretes digestive enzymes into the gut through the pancreatic duct system, the islets of Langerhans secrete endocrine hormones into the blood. Through four decades of research, it has become clear that islets are not just unstructured endocrine cell aggregates but rather highly organized micro-organs. They have species-specific three-dimensional architecture, which is critical to their proper function in response to nutritional stimuli. Islet architecture facilitates endocrine cell polarity and connection with the microvasculature to guarantee secretion of insulin into the capillaries, physical and electrical cell-cell coupling to assure synchronous hormone secretion, and directionality of intra-islet paracrine signaling and connection with the nervous system for feedback regulation.^1–4^ Islet architecture is disrupted in all types of diabetes mellitus.^5,6^ It is also disrupted in cadaveric islets during isolation and culture prior to islet transplantation as well as after infusion into the portal vein, limiting transplantation outcomes.^7–9^ Moreover, recapitulating native islet architecture remains a key challenge for *in vitro* generation of islets from stem cells.^10^

Recent research has focused on how three-dimensional islet architecture forms during development, and how it is maintained during the tissue remodeling processes that accompany islet adaptation to life events such as pregnancy and obesity. Research has also investigated how islet architecture is re-formed during regeneration after injury and how it is lost in diabetes. In this review, we discuss work that has led to the current understanding of determinants and dynamics of pancreatic islet architecture. We further discuss how islet architecture influences islet function and how mathematical modeling approaches can help to investigate this structure-function relationship.

## Interspecies comparison of islet architecture

**Figure 1.**
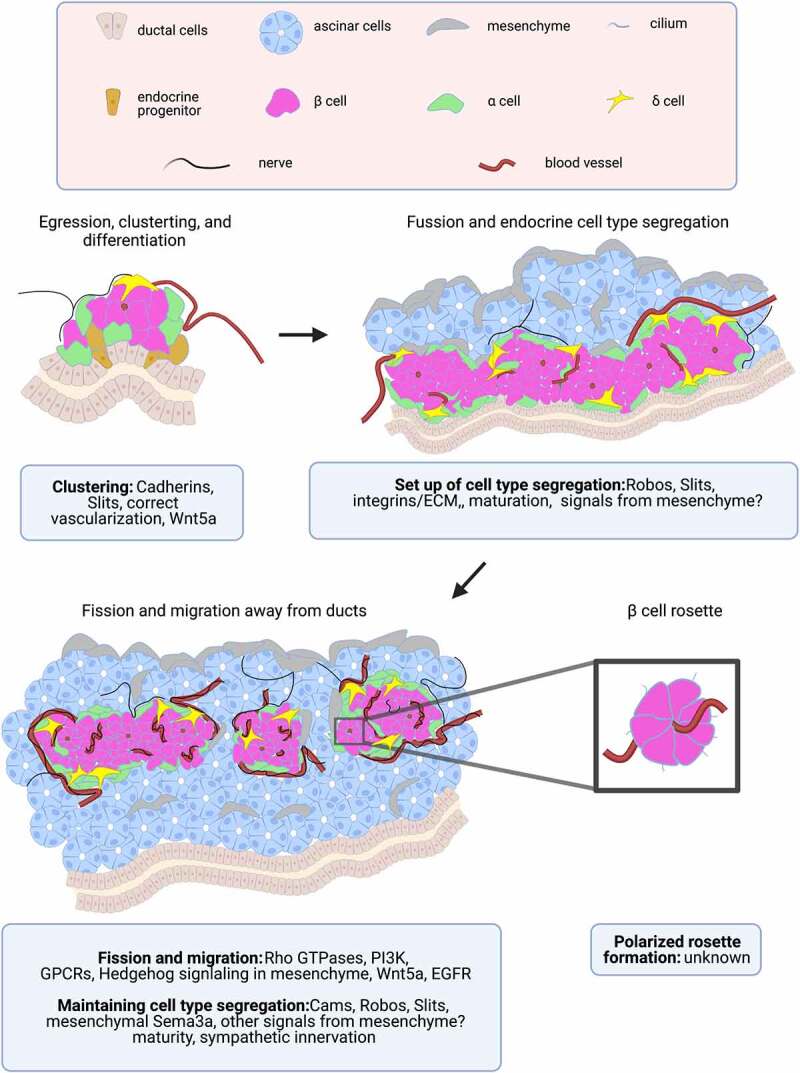
**Stages and determinants of islet morphogenesis:** Egression, clustering, and differentiation: Neurog3 high endocrine progenitors egress from the bipotential epithelial cord in clusters that differentiate into islet endocrine cells while remaining attached and adjacent to this cord. Nerves and blood vessels closely associate with these clusters early on, possibly providing morphogenetic signals. Fusion and endocrine cell type segregation: Endocrine cells expand and fuse to adjacent clusters, creating long strings of primitive islet structures along the ducts. These structures have segregated endocrine cell types and are surrounded by extra-islet nerves, ascinar tissue, and mesenchyme, which provide morphogenetic signals. Blood vessels now penetrate the islet via mutual attraction of endothelial and β cells. Fission and migration away from ducts: Elongated islet structures and size of adult islets. Extra-islet nerves have invaded the islet through scaffolding with intra-islet vasculature, and β cells have reached maturity. Islet endocrine cell type segregation is maintained throughout homeostatic adulthood. β celll rosette: β cells within the islet organize into rosette structures around blood vessels with their cilia projecting into the extracellular space, though timing and morphogenesis of this organization is unknown. Figure created by Biorender.com.

Stereotypical mouse islet architecture consists of a rounded core made mostly of insulin-secreting β cells, while glucagon-secreting α cells, somatostatin-secreting δ cells, and pancreatic polypeptide-secreting PP cells are restricted to the islet periphery^11^ (see Figure 1 below). β cells within the core of the islet form polarized rosette structures surrounding blood vessels.^12^ Within rosettes, β cells have an apical domain located on the outside edge of the rosette where primary cilia project into shared extracellular spaces, a basal domain next to the blood vessel where the nucleus and docked insulin granules lie, and lateral domains between β cell edges enriched in glucose transporters and Ca^2+^ sensing machinery.^12–15^

Human islet architecture is more complex, with several different proposed models for the “stereotypical” human islet.^16–19^ The ratios between the main endocrine cell types in the islet are also different between mice and humans, with approximately 70% β cells, 20% α cells, and <10% δ cells in rodents^20^ compared to approximately 60% β cells, 30% α cells, and 10% δ cells in humans.^16,17,21,22^ The differences between the various models of human islet architecture and its resemblance to the stereotypical rodent islet may have arisen from differences in qualitative and quantitative approaches to describe it^23^ or that human islets do not have a stereotypical architecture at all.^24^ Nevertheless, evidence supports the existence of a conserved design principle between these two species, where homotypic interactions among endocrine cells of the same type are prioritized over heterotypic interactions between endocrine cells of different types.^24–26^ Recently, it was also shown that there are conserved β cell polarity domains between mice and humans, though the actual functional roles of these domains in islets is an area of active research and remains to be fully elucidated.^14,15,27,28^

Several comparative studies involving species beyond mouse and human suggest that though islet endocrine cells can organize in many ways across species, this may be due more to differences in physiological demand rather than developmental mechanisms.^6,11,29^ Islet architecture can be very different even in closely related species like cats and hyenas, while more evolutionarily distant species like elephants and rabbits can share strikingly similar organizations.^6^ Further, species with different architectures like human, monkey, pig, rabbit, mouse, and bird have similar islet size distributions, suggesting a common developmental mechanism. Even more, islet architecture is mutable within species if physiological demands change (see “Changes in islet architecture during adult life” section below). Studies in a variety of animals models other than rodent, where physiological perturbations are introduced, will help clarify the relative contribution of developmental mechanisms and physiological demands on islet architectural differences between species.

## Formation of islet architecture during development

Pancreatic endocrine cell differentiation and islet formation during embryonic development have been thoroughly covered elsewhere and will not be discussed in detail here. For detailed review of islet development, see references.^30–33^

In brief, islet morphogenesis begins with transient high expression of the transcription factor Neurogenin3 (Neurog3) in individual bi-potential progenitor cells of the pancreatic trunk epithelium around embryonic day (E)13.5 in mice, which designates them as endocrine progenitors.^34–36^ Until recently, the prevailing dogma maintained that Neurog3+ endocrine progenitors delaminate from the pancreatic epithelial cord and migrate through the mesenchyme as single cells, simultaneously undergoing differentiation and eventually clustering to form mature islet architecture away from the ducts.^30,31^ This idea was initially supported by the observation that islets are polyclonal in nature and thus come from multiple founder endocrine progenitors.^37^ However, recent work has provided compelling evidence that endocrine progenitors instead remain adjacent and attached to the epithelial cord throughout endocrine cell differentiation and most of islet formation.^38,39^ Further, there is evidence that islets initially develop as long interconnected chains along the ductal epithelium and only resolve into the rounded distinct structures of the adult through fission and subsequent engulfment of the islet by surrounding mesenchyme.^40^ Interestingly, it appears that human islets develop first with a mantle-core architecture like that of rodent islets, which is replaced by the intercalating α-β cell architecture of the human islet during the first months of postnatal life.^41,42^ Though there have been several hypotheses put forth to explain the formation of the classic core-mantle organization of the endocrine cell types in developing islets, an actual mechanism remains elusive.^38,43^

## Determinants of islet architecture

There is a large body of research that describes molecules which affect the development and maintenance of islet architecture. Here we summarize what is known about determinants of islet architecture in hopes of gaining insight into a more unified view of the underlying mechanisms driving islet morphogenesis and maintenance.

### Cell–cell and cell–matrix adhesion molecules

Rodent endocrine cells can spontaneously self-organize *in vitro* into stereotypical core–mantle architecture when they are re-aggregated after dispersion, likely due to intrinsic differential cell–cell adhesion properties.^44^ Interestingly, however, when human islets are dissociated and allowed to re-aggregate, they tend to form inverted core–mantle architecture such that α cells form the core and β cells form the mantle, indicating that intrinsic cell–cell adhesion properties in human endocrine cells may not be sufficient to fully recapitulate *in vivo* architecture.^7,45^ This is further supported by the observation that human islets transplanted back into the *in vivo* environment undergo further architectural changes that resemble human like morphologies.^7^ It is becoming increasingly clear that cell–cell adhesion molecules are necessary for endocrine cell clustering and maintenance of islet cell type segregation during islet morphogenesis and throughout life. One family of these molecules, the Cadherins, has been shown to be important for islet endocrine cell clustering. Blocking normal localization of Cadherins in general *in vivo* via expression of a dominant-negative E-cadherin in β cells prevents normal clustering of endocrine cells at E18.5 in mice. However, deletion of single Cadherins leaves islet architecture intact suggesting that Cadherins can compensate for each other in this role.^46–49^ In addition to controlling cell clustering, there is evidence that Cadherins and cell adhesion molecules (CAM) are important for maintaining cell type segregation in the islet. It is thought that differential expression of Cadherins and CAMs between different cell types in the islets creates differential tissue surface tensions, which in turn guide self-segregation of the cells to their appropriate locations.^50–52^ Particularly, NCAM knockout mice show some intermixing of α and β cells starting at 4–5 weeks of age, while earlier developmental time points remain unaffected. This defect in islet architecture was attributed to a redistribution of Cadherins to the center of β cell rosette-like structures instead of lateral domains of individual β cells, supporting a role for correct adherens junction localization in maintenance of islet architecture.^53^ Unlike NCAM, the cell adhesion molecules Roundabout (Robo) receptors were shown to be necessary for the development of islet architecture as early as E18.5 in mice. When *Robo1* and *Robo2* genes are deleted selectively in β cells in either early or late stages of islet morphogenesis, endocrine cell type segregation is severely disrupted, while β cell identity remains unaffected.^54^ Importantly, deletion of *Robo2* in α cells has no effect on islet architecture, implying that β cells themselves are the main endocrine determinant of islet architecture.^54^ This is further emphasized by the observation that eliminating α cells altogether had little effect on β cell clustering and overall islet formation.^55^ Importantly, Robo is the only identified factor to date that governs the development of islet architecture uncoupled from β cell differentiation.^54^

The extracellular matrix (ECM) is an important molecular and mechanical signaling hub that changes both its repertoire of signaling molecules and its relative stiffness throughout time and space to influence cell behaviors in a variety of developmental processes.^56^ Thus, it is not surprising that the ECM and cell–matrix adhesions molecules are also implicated in islet morphogenesis. Specifically, α_v_ integrins are differentially expressed in fetal and adult mouse pancreatic endocrine cells, and can control differential adhesion to ECM components present in the developing and adult pancreas.^57^ Supporting a role for integrins in controlling islet architecture, when human fetal pancreatic tissue explants were implanted in the kidney capsule of mice treated with an integrin blocking peptide, islets remained closely associated to the ducts, with their α and δ cells specifically oriented toward the duct.^58^ This bipolar islet architecture is reminiscent of the fetal islet architectural stage observed early during islet morphogenesis in humans, suggesting that differential adhesion of endocrine cells to matrix molecules may affect islet architecture development in humans.^59^ In support of this, a recent matrix-specific proteome analysis of human fetal and adult pancreatic tissue showed age-dependent differential expression of many ECM and matrix adhesion molecules implicating them as possible regulators of human islet architecture.^60^ Thus, changes in expression of ECM components and matrix adhesion molecules such as α_v_ integrins throughout development likely govern islet architecture formation during development and possibly in adulthood.

### Cytoskeletal Regulators

During islet morphogenesis, endocrine cells undergo active cytoskeletal remodeling in part to facilitate separation of islets into discrete units away from the ductal epithelium.^61,62^ In particular, the Rho GTPase family of cytoskeletal regulators has been shown to mediate this aspect of islet morphogenesis. β cell-specific expression of a dominant negative form of the Rho GTPase Rac1 in mice resulted in larger islets which remained close to the duct.^63^ Similarly, the Rho GTPase Cdc42 was shown to affect islet morphogenesis through its ability to modulate N-WASP, which ultimately controls the dynamics of junctional F-actin complexes between neighboring cells. Disrupting Cdc42 activity in mice results in improper delamination of newly formed β cells from the ductal epithelium and dedifferentiation of β cells in adult islets, both of which are partially rescuable by N-WASP knockout.^62^

Disruption of proteins that regulate Rho GTPases cause similar defects in islet morphogenesis. When PI3K, an upstream regulator of Rac1, is inhibited in zebrafish, islet cells fail to migrate and coalesce to form the primary islet normally found in zebrafish.^61,64^ This was attributed to abrogation of filopodial extensions and loss of directional migration, like the phenotypes observed in Rho GTPase mutant mice. Confoundingly, this same phenotype is not recapitulated when Rac1 is knocked down with a morpholino in zebrafish, which may be due to compensatory signaling from other small GTPases or to species-specific differences in islet morphogenesis between mice and zebrafish.^61^

Altogether, the body of work on cytoskeletal regulation during islet morphogenesis points to a role in the formation of discrete islet units and migratory behavior during neonatal islet development.

### Mesenchyme

The pancreatic mesenchyme affects islet morphogenesis. When pancreatic mesenchyme is severely overgrown due to unregulated mesenchyme-specific Hedgehog signaling in mice, stereotypical islet architecture is replaced by a heterogeneous mix of architectures, in which some islets appear normal, some are intermixed, and some have inverted core–mantle organization.^65^ However, it has yet to be determined whether these architectural phenotypes are due to the overgrown mesenchyme itself or to the mis-regulation of Hedgehog signaling in this mouse model. Confoundingly, it was found that when mesenchyme is ablated at different developmental time points from E9.5 to E16.5, using an inducible diphtheria toxin (DT)-based ablation driven by the mesenchyme marker Nkx3.2, pancreas endocrine mass is reduced but islet endocrine cell ratios and architecture remain intact, suggesting that islet architecture development is not completely dependent on signals from the mesenchyme.^66^ However, recent evidence suggests that pancreatic mesenchyme is not homogenous but rather composed of several subpopulations that may be spatially and functionally distinct *in vivo*.^67,68^ Thus, it is possible that subpopulations of mesenchymal cells not descended from the Nkx3.2 lineage may be present, and if so, could be sufficient for mesenchymal maintenance of islet architecture.^67–69^

Specific factors from the mesenchyme that affect islet morphogenesis have been identified. In the pancreas, *Hox6* is expressed specifically in the mesenchyme and upon its genetic deletion, mice show a large reduction in cell mass of all endocrine cell types, which can be rescued in explant culture by exogenous Wnt5a.^70^ Semaphorin 3a (Sema3a), which binds the Neuropilin 2 (Nrp2) receptor in islet endocrine cells, is expressed in a gradient within the pancreatic mesenchyme such that high levels are present at the edge of the organ, while lower levels are present toward the center. This Sema3a gradient is thought to attract endocrine cells away from the duct during development, as evidenced by cell migration assays and disrupted separation and migration of islets away from the ductal epithelium in *Nrp2^−/ −^* mice.^71^ Whole-body knockout of the Robo Receptor ligands, *Slit2* and *Slit3*, which are expressed in the pancreatic mesenchyme but not in the islet, results in severe defects in islet cell clustering at E18.5.^72^

Some factors expressed in both mesenchyme and other pancreas tissue compartments affect islet morphogenesis, but their tissue-specific roles have yet to be untangled. Wnt5a is expressed throughout the fetal pancreas including the mesenchyme, while its receptor Fzd2 is restricted to the endocrine compartment by late developmental stages. Whole body knockout of *Wnt5a* in mice results in elongated islets that remain closely opposed to the ductal epithelium, while over expression results in small, scattered islets.^73,74^ Similarly, EGFR is expressed throughout the pancreas, including the mesenchyme, while its ligand TGF-α is expressed in β cells in humans and mice. Whole body knockout of *EGFR* in mice shows defects in islet fission and migration away from the ducts.^75^ TGF-β is also expressed in the pancreatic mesenchyme and the endocrine compartment, and mouse embryonic pancreas organ explants treated with pan-TGF-β inactivating antibodies ceased islet budding.^76^ Some experiments have been performed to untangle the tissue specific roles of TGF-β in the pancreas, though these have not been entirely elucidated. For example, expression of a mesenchyme specific dominant negative TGF-βRII causes a hyper-proliferation of endocrine cells though no defects in morphogenesis *per se* are observed.^77^ Additionally, over expression of the TGFβ signaling inhibitor Smad7 in Pdx1 expressing cells from birth results in a 90% reduction of β cells and islets made of mostly α cells.^78^

Both the EGFR and the TGF-β-dependent islet morphogenesis phenotypes described above were originally attributed to changes in activity of the matrix metalloproteinases MMP9 and MMP2, respectively. Accordingly, it was shown that treatment of embryonic pancreas explants with a specific or a general MMP inhibitor recapitulates the phenotype seen in the pan-TGF-β inactivating antibodies treatment.^76^ However, *MMP2/9* double knockout mice undergo normal islet morphogenesis, and when *TIMP1*, a modulator of many MMPs, is knocked out or over expressed, islet morphogenesis proceeds normally. This supports the idea that MMPs are dispensable for islet morphogenesis *in vivo*, however a role for other redundant MMPs, or of MMPs not under regulation by TIMP1, cannot be ruled out.^79^ Indeed, it was shown that when *MMP9* is deleted in mice, expression of MMP7 increases in the islets suggesting compensation by other MMPs is possible.^80^

Though it remains unclear if the mesenchyme is fully required for normal islet architectural development, signals from the mesenchyme can and do influence islet architecture and islet morphogenesis more generally.

### Vasculature

Islet vasculature and endocrine cells reciprocate signals during islet morphogenesis. It was hypothesized that endothelial cells attract endocrine cells, causing the latter to migrate through the mesenchyme and coalesce around blood vessels to form the islets.^81,82^ However, β cells can themselves attract vasculature via the production of VEGF-A.^82,83^ An extreme example of this is seen in experiments in which acinar tissue is reprogrammed directly into β cells in mice pancreata *in vivo* by viral infection of the transcription factors *Neurog3, Pdx1*, and *MafA*.^84^ In these pancreata, the newly reprogrammed β cells, initially present as single β cells scattered across the tissue, secrete VEGF, and remodel the pancreatic vasculature by attracting new blood vessels to themselves.^84^ Strikingly, the newly reprogrammed β cells migrate toward each other over time and aggregate to form “islet-like” clusters, attracting both close vascular support and innervation.^85^

Nevertheless, over expression of VEGF-A in β cells during the embryonic and postnatal periods in mice results in reduced endocrine cell migration from the duct, and almost completely abolishes islet morphogenesis, indicating that too much vasculature is deleterious to islet development.^86^ It is possible that control of the correct amount of VEGF-A in β cells during development is mediated by the β2-adrenergic receptor, Adrb2, which suppresses VEGF-A production in β cells. Deletion of *Adrb2* in pancreatic endoderm during mouse development results in increase in the proportion of small, hyper-vascularized, islets, though no change in overall islet architecture was seen.^87^ Partial reduction in islet vasculature down to approximately 30% of its normal level, on the other hand, has little effect on formation of islet architecture in either mice or zebrafish embryos.^88,89^ However, near complete elimination of islet vasculature in zebrafish embryos resulted in disrupted islet architecture.^90^ Interestingly, in experiments in adult mice, islet architecture was altered following β cell death in an inducible model of VEGF-A over expression but was restored after β cell regeneration following withdrawal of the VEGF-A induction.^91^

### Innervation

Sympathetic pancreatic innervation, which closely follows vasculature during development,^92,93^ also mediates islet architecture. When sympathetic nerves are genetically ablated in mice, the ratio of islets with α cells found in their core increases compared to normally innervated controls.^94^ Since β cells in these mice show maturity defects, it is unclear if the disrupted cell type segregation is caused directly due to loss of morphogenetic signals from the nerves, or indirectly through the loss of β cell maturation (see next section). Additionally, islets in these mice remain closer to the ducts and have elongated morphologies reminiscent of islets in mouse models with cytoskeletal and cell migratory defects. It was proposed that these cell migration defects in islets of mice with genetically ablated sympathetic nerves are caused by directional adrenergic signaling from neurons to β cells, as β cells migrate toward sympathetic ganglia in coculture, but not when an adrenergic antagonist is added to the media.^94^ Additionally, the islet architecture defects in these mice are partially rescuable *in vivo* by treatment with a β-adrenergic agonist. Interestingly, it was shown that when just the intra-islet nerve fibers, which normally develop in the first week of life, are ablated, islet architecture remains intact, indicating that intra-islet but not extra-islet pancreatic innervation is dispensable for normal islet architecture.^88,92^

### Maturation and endocrine cell Identity factors

One of the strongest determinants of islet architecture is β cell maturity. Between birth and weaning, rodent β cells undergo a maturation process that results in acquisition of the adult glucose stimulated insulin secretion response.^95–97^ To date, virtually all experiments in which mouse β cells were prevented from reaching their mature identity or were forced to lose it after it had been acquired showed some degree of concomitant loss of canonical core-mantle architecture, marked by intermingled α cells in the islet core. Preventing β cells from assuming mature identity during development by genetic deletion of the key transcription factors *Pdx1, MafA*, or *NeuroD1* results in partial or complete loss of canonical mouse islet architecture.^98–101^ Similar results are seen in transgenic mice over-expressing the Nkx2.2-repressor domain in mature β-cells.^102^ This was also seen with over expression of a dominant-negative form of HNF-1α, which was associated with reduced expression of E-cadherin in the immature β cells.^103^ Likewise, when expression of the β cell progenitor transcription factor, *HNF6a* is forced past its normal temporal domain (after E18.5), β cells fail to mature and islets lose endocrine cell type sorting.^104^ The loss of endocrine cell type sorting following continued expression of HNF6a may be due to the subsequent decrease in expression of CTGF, as mice with *CTGF* deletion show intermixing of endocrine cell types in adult islets.^105^ Additionally, when *mTOR* is deleted in β cells before architecture develops, β cells fail to mature and islets remain elongated and close to the ducts.^106^ Other factors for which genetic disruption resulted in both loss of β cell maturation and loss of canonical islet architecture include DICR1 (when deleted in embryonic β cells),^48^ Synaptotagmin,^107^ αCatenin,^108^ and, to some extent, BMPR1α.^109^

Even when β cells reach maturity and architecture has formed normally, loss of β cell maturity in adulthood still results in loss of normal islet architecture. For example, deletion of *MafA* in adult mice β cells results in a delayed loss of β cell identity markers by 8 weeks of age. Lagging just behind this loss of maturation, at 12 weeks of age severe intermixing of endocrine cell types, is observed.^110^ Similarly, when the maturation associated transcription factor *Insm1* is deleted in β cells in the adult mouse, β cells lose maturity markers, and α cells are subsequently found mixed into the islet core.^111^ Interestingly, even in islets of healthy adult mice, a sub-population of immature β cells located in the periphery has been reported to migrate into the islet core concomitant with gaining mature gene expression and glucose responsiveness,^112^ suggesting a connection between the degree of β cell maturation and their location within the islet.

However, it remains to be determined if β cell maturation is required for correct islet architecture, and/or if correct islet architecture itself is required for proper β cell maturation.

## Changes in islet architecture during adult life

Once islet architecture has been established, it does not remain static throughout life. Rather, various physiological stresses like obesity, diabetes, and pregnancy result in architectural remodeling of the adult islets. This plasticity of adult islet architecture suggests that there may be a functional role for islet architecture remodeling. However, whether these changes are adaptive or pathological remains largely unknown. The following sections will discuss what is known about architectural changes in islets in adulthood.

### Islet architecture in successful compensatory islet expansion

During times of increased metabolic demand, such as in mild obesity, the islets of Langerhans expand to compensate for the increased need for insulin, while retaining their correct spatial endocrine cell type positioning.^113^ Similarly, during pregnancy, islets undergo β cell expansion, adapting to the transient insulin resistance in the pregnant female, while subsequent to pregnancy a retraction of β cells to pre-pregnancy levels occurs.^114^ Concomitant with this β cell expansion, transient architectural remodeling may occur.^115^ Short-term induction of islet mass expansion in mice by acute administration of the insulin receptor antagonist S961 or glucagon receptor antagonists causes large increases in islet size, but the expanded islets retain their typical mantle-core architecture.^116,117^ Thus, in successful compensatory islet mass expansion, islets retain their three-dimensional architecture. How islets maintain correct spatial architecture while undertaking the massive tissue remodeling required for compensatory expansion remains an open question.

### Islet architecture in type 2 diabetes

Islet architecture is disrupted in mouse models of type 2 diabetes (T2D), yet how and why this happens remains unknown.^118^ There is evidence that the observed disruption in islet architecture found in models of T2D is in part due to transdifferentiation of β cells that already populate the islet core, rather than an invasion of α and δ cells from the islet mantle.^119^ For instance, in several mouse models of T2D that display disrupted islet architecture, including *db/db*, GIRKO, and metabolically stressed *FoxO1*-knockout mice, a majority of the loss in β cell mass is due to β cell dedifferentiation rather than β cell death, and in some cases, these dedifferentiated β cells partially transdifferentiate to other endocrine cell types.^119^ Similarly, diabetic *insulin*-knockout mice also show islet architecture abnormalities, with strong β cell dedifferentiation observed after 34 days of severe diabetes.^120^ These observations suggest that under physiological stresses like diabetes, mechanisms that maintain islet architecture during compensatory expansion may be perturbed. Interestingly, most dedifferentiated β cells in these models do not undergo a full transdifferentiation to other endocrine cell types. This could suggest that in diabetes, architecture is disrupted because incomplete lineage transition may not allow for expression of molecules regulating cell sorting appropriate to their “new” cell type. Other rodent models of diabetes in which islet architecture is altered include diabetic Goto-Kakizaki rats, mice with a β cell-specific deletion of the gene *fumarate*, and mice with mutated version of K_ATP_ channel (βV59M).^5^ Finally, islet architecture in T2D can be disrupted also by deposition of human islet amyloids.^121^

In some contexts, architectural disruptions in the adult mouse due to T2D are reversible. For example, Brereton and colleagues showed that hyperglycemia induced by *Kir6.2* activating mutations in mice led to massive β cell identity loss and expansion of α cells in the islet core. When hyperglycemia was rescued with insulin or sulfonylurea pellets, the islet architectural defects and α cell mass increase were rescued, indicating that restoration of β cell identity could restore normal islet architecture in the adult.^122^ Similarly, work from Cheng and colleagues suggests that in diabetic *db/db* mice, treatment with a fasting-mimicking diet rescues diabetes induced defects in islet architecture, although these changes were not specifically quantified.^123^ It remains unknown if the rescue of islet architecture in these models is due to a passive or active sorting processes, *i.e*., whether incompletely transdifferentiated β cells in the core of the islet are restoring their identity, or rather new non-β endocrine cells are actively self-segregating from the β cell core when normoglycemia is restored.

### Islet architecture in type 1 diabetes

In mouse models of type 1 diabetes (T1D) where β cells are largely killed by an immune attack, the mechanism of islet architecture disruption may differ from that of models of T2D where β cell death is likely not a large contributing factor in the early stages of disease development. In non-obese diabetic mice (NOD), a model of immune mediated T1D, islet architecture appears to be largely disrupted due to β cell core collapse after immune cell invasion. This likely clouds the ability to see any real architectural changes since the resulting islets are mostly comprised of non-β endocrine cells intermixed with lymphocytes.^115^ Nevertheless, in studies of human islet architectural defects in early and late stage T1D patients, it appears that immune cell invasion within the core of the islet is highly variable between donors, yet architectural defects are ubiquitous. Thus, these defects are likely caused by additional factors other than massive immune infiltration.^124,125^ As T1D progresses, β cell mass decreases while α cell and δ cell masses are less affected, and by late stages of T1D almost all hormone-positive cells within T1D stain positive for glucagon or somatostatin.^124,126^ Additionally, many glucagon-positive cells in human T1D islets also stain positive for β cell markers like Nkx6.1, which could possibly be due to β to α cell transdifferentiation.^124–126^ Hormone-negative, immune cell marker-negative clusters of cells are also found interspersed throughout human T1D islets, which are thought to be degenerative β cells.^125^ Thus, it is plausible that, as in T2D, transdifferentiation and loss of β cell identity and maturity are also contributing factors to the abnormal architectures observed in T1D.

### Islet architecture in β cell regeneration

Three models of β cell ablation, namely, severe ablation of all β cells by β cell-targeted diphtheria toxin (DT) expression, a less acute β cell targeted DT that destroys 70–80% of β cells, and Alloxan injection, provide insight into how β cell regeneration might affect islet architecture. In all three of these ablation models, islet architecture is disrupted, yet each differs in its ability to regenerate normal islet architecture after restoration of normoglycemia.^123,127–129^ Thorel and colleagues reported that in acute β cell ablation with DT where all β cells were destroyed, 10% of the β cell mass can be regenerated with periodic insulin treatment, mainly by transdifferentiation from α cells.^128^ However, in this model, islet architecture appears to remain disrupted even 10 months after the start of regeneration, though this was not specifically quantified.^128^ On the other hand, Nir and colleagues performed less acute β cell ablation via a β cell specific DT protocol that only destroys 70–80% of β cells.^129^ In this model, β cell regeneration was derived mostly from replication of surviving β cells, and the resultant islets had a near-perfect architecture 23 weeks into the regeneration process.^129^ These seemingly contradicting results may be due to the different origin of the regenerating β cells (transdifferentiation *versus* existing mature β cells). However, this may not be the only explanation, as when Alloxan treated mice were infected with a virus that forced expression of *MafA* and *Pdx1* in α cells to convert them to β cells, normal architecture was restored though this was also not specifically quantified.^127^ The difference in ability to restore normal islet architecture upon restoration of normoglycemia could be due to the difference in the source of new β cells in each of these models. In the acute DT and Alloxan ablation model, new β cells come from transdifferentiated α cells. The new β cells that formed from forced expression of *MafA* and *Pdx1* in the Alloxan model were shown to be fully mature and likely relatively transcriptionally similar to a normal β cell, while the α cells transdifferentiated in the acute DT model were less committed to the β cell fate.^123,127,128^ In the more mild DT model from Nir and colleagues, a pool of surviving β cells provides the source for new β cells.^129^ Taking these three models together, this suggests that β cells arising from naturally transdifferentiating α cells, as is the case in the acute DT ablation model, may be less committed to the β cell lineage and thus not express the necessary factors important for endocrine cell type segregation. However, when new β cells that are more similar to fully mature β cells are generated, like in the case of forced expression of *Pdx1* and *MafA* in the Alloxan treated mice or from the pool of existing β cells as is the case in the milder DT model, they may be transcriptionally closer to native β cells and thus express the full array of factors necessary for generating correct islet architecture.

### Islet architecture in cultured and transplanted islets

Islet architecture and tissue integrity are disrupted in human cadaver islets during isolation and culture prior to islet transplantation, as well as after infusion into the portal vein during islet transplantation treatments in patient with T1D.^7–9,130^ During the culture period after islet isolation, human islets lose preferential homotypic interactions between β cells and display a more random cell arrangement.^7^ However, 2 weeks after transplantation of isolated human islets to the kidney capsule or in the liver of mice, islets regain more human-like architecture, with subunits of β cells surrounded by α cells that line newly formed blood vessels.^7^ Even more, when dispersed human islet cells are allowed to reaggregate *in vitro*, they form an α cell core with a β cell mantle, yet after 2 weeks post kidney capsule or liver transplantation, they display normal human islet architecture with β cell cores and α cell mantles lining the vessels.^7^ It is yet unknown what causes these changes in islet architecture in culture and after transplant, but a possible mechanism may be related to interactions between endocrine cells and matrix proteins secreted from vessels.^7^

In some cases, human islet-like clusters derived *in vitro* from human pluripotent stem cells were reported to re-organize after transplantation under the kidney of mouse recipients. These transplants were described as either having typical rodent-like islet architecture^131^ or human-like architecture.^132^ Similarly, transplantation of clusters of earlier stage progenitor cells derived from human pluripotent stem cells gave either rodent-like^133^ or human-like architecture.^134^ Fetal human pancreatic islets transplanted into mice were reported to mature into human-like islet morphology.^135^ However, to date, no systematical morphological quantifications were reported of the architecture of stem cell-derived islet-like clusters after transplantation, and it is evident that either of the grafts do not show the overall morphology of *bona fide* islets. These discrepancies will be resolved with more thorough quantification of islet architecture, which was not in the scope of the above works.

## Relationship of islet architecture to islet function

Islet architecture is important for normal islet function. Homotypic interactions between β cells, allowed for by β cell clustering within an islet, are required for the normal synchronous oscillations between β cells which underly controlled glucose stimulated insulin secretion (GSIS). This is evidenced by the observation that dispersed β cells with no cell contacts display heterogeneous uncoordinated oscillations at both basal and elevated glucose levels, which results in high insulin secretion at the basal glucose level and low uncoordinated insulin secretion at the high glucose levels.^136^ This is because one of the most important mechanisms through which β cells synchronize GSIS is via electrical coupling using Connexin36 (Cx36) gap junctions that form between touching β cells, which allow for exchange of the cations that ultimately control these oscillations.^137–139^ In addition to clustering between β cells, the spatial organization of heterogeneous β cell subpopulations within an islet is also important for normal GSIS. Electrically coupled β cells within an islet display varying levels of excitability and metabolic rates which help to suppress calcium oscillations throughout the islet at low glucose and regulate calcium oscillation dynamics at high glucose.^140^ These β cell subpopulations characterized by varying levels of excitability and metabolic rate display a nonrandom spatial organization across the islet that is likely important for GSIS dynamics.^4^ Specifically, it was shown that glucose induced Ca^2+^ wave initiation sites, an intrinsic and stable feature of an islet, occur in areas with both low excitability and metabolic rate and thus a faster intrinsic oscillation frequency, a quality that has been suggested to promote wave origination.^4,141^ Recent work using *in vivo* imaging of Ca^2+^ dynamics in the Robo βKO mouse model has further highlighted the importance of 3D cell organization on islet function beyond simple clustering. Robo βKO islets show normal endocrine cell clustering and maintain intrinsic β cell identify and function yet display extensive endocrine cell type intermixing resulting in decreased β cell homotypic interactions.^54,142^ These features make this model uniquely suited for testing the effects of 3D endocrine cell organization on islet function, uncoupled from β cell pathology. Intravital Ca^2+^ imaging revealed that Robo βKO mice display a decrease in synchronous oscillations between intra-islet β cells, that is reminiscent of the reduced oscillations synchronicity phenotype seen in islets from mice heterozygous for the *Cx36* gene (*Cx36^+/*–*^*).^137,139,142^ However, unlike *Cx36^+/–^* islets, Robo βKO islets show no change in *Cx36* gene expression or Cx36 plaque area, and Cx36 gap junctions still localize to their normal domain at the β cell borders. This indicates that disruption in synchronous oscillatory behavior in Robo βKO islets is likely due to changes in islet architecture itself rather than decreased expression or mis-localization of gap junction machinery.

In addition to electrical β cell-β cell coupling, islet architecture also influences directionality of diffusible paracrine and autocrine signals within the islet, and these in turn affect islet function.^143–148^ Furthermore, the spatial architecture of the islet facilitates direct receptor-ligand contacts between adjacent β cells that influence GSIS dynamics independent of electrical coupling. Indeed, dissociated wild type β cells have worse GSIS response than that of intact islets lacking Cx36 gap junctions.^149^ One such mechanism of non-electrical regulation of GSIS dictated by cell-cell contacts is that of EphA-ephrinA binding between β cells.^150^ Finally, islet architecture also influences β cells electrical coupling to neighboring δ cells, which can add another layer of regulation to hormone secretion.^151^

Further highlighting the importance of 3D islet organization, a recent report found that induced pluripotent stem cell (iPSC) derived islets become more functionally mature when subjected to a maturation step during *in vitro* differentiation that causes changes in islet architecture resembling those found during human islet morphogenesis.^152^ This transition to a mature human-like islet architecture was not accompanied by an increase in β cell mass yet was still correlated with lower basal insulin release and increased glucose stimulated insulin secretion compared to islets that had not undergone this maturation stage. Further, a pseudo-time analysis of single cell RNAseq data that looked at iPSC derived islets during the functional *in vitro* maturation step, during *in vivo* engraftment which causes further maturation, and in adult islets, found that Robo1 and Robo2 levels were highly dynamic across these stages. Specifically, Robo1 and Robo2 were expressed at high levels during the attainment of mature-like architecture and lower in the adult islet, consistent with their role in regulating the development of normal islet architecture important for controlled GSIS.^152^

It has been hypothesized that insulin secretion is not only synchronous within an individual islet but across all islets in the pancreas and thus is responsible for pulsatile insulin levels in circulating blood observed in humans and mice.^153^ Though this remains to be rigorously tested, if it proves true this is likely a very important function of synchronous oscillations within islets. Peripheral insulin pulsatility is important for mounting a robust response to insulin in the liver, for keeping peripheral tissues insulin sensitive, and for allowing the readily releasable pool of insulin granules time to replenish during sustained glucose stimulation.^153–157^ Pulsatile insulin levels are also known to be disrupted early in diabetes disease progression, often before overt diabetes has set in.^153,157^ Thus, it is an interesting possibility that the architectural defects seen during diabetes progression may contribute to the perturbations in pulsatile insulin secretion observed in mouse models of diabetes and pre-diabetic and diabetic patients.

### Using modeling to understand islet architecture-function relationship

Computational modeling also provides support for the idea that islet architecture affects islet function. Nitalla and colleagues created models of oscillating β cell clusters to demonstrate that higher levels of coupling between β cells allow for higher resistance to pathological perturbations like loss of β cells from diabetes.^158^ Hoang and colleagues further expanded on these models by including α cells and using experimentally derived parameters to define the intrinsic ability of endocrine cells to oscillate, including effects from autocrine and paracrine interactions.^159^ Using this model, they tested how artificial arrangements of α and β cells oscillate at varying glucose levels and found that both phase and synchronicity were dependent on specific architectures. Even more, they found that the specific cell ratios of mouse and human islets are likely important for handling dynamic glucose changes in their respective architectures. When diabetes induced changes to islet architecture were simulated by removing β cells at random within islets, synchronization of β cells decreased at high glucose levels as β cells were removed, even when islets were allowed to re-aggregate after 50% of β cell loss.^159^ In another example of modeling the relationship between islet architecture and function, Sterk and colleagues used mathematical modeling and confocal imaging of mouse pancreatic tissue slices to show that the origin of calcium waves are primarily triggered by a specific region of the islet.^160^ As more sophisticated methods to quantify and model human islet architecture emerge,^161^ it will be interesting to see what new information will be gained on the role of islet architecture in islet function.

## Concluding remarks and open questions

Accumulating data indicate that islet architecture is important for optimal islet function. Through mouse genetics and mathematical modeling in human islets, we are beginning to understand what parts of islet architecture are important for which functional aspect of islet biology, from optimal hormone response to stimuli, to β cell maturation and identity. At the same time, new data and models allow us to decipher different cellular and microenvironmental determinants controlling islet architecture. In the future, these could be used to optimize and impose correct architecture on islet-like clusters made *in vitro* from human pluripotent stem cells, and perhaps also to target islet architecture as another layer of control in helping islets adapt to different diabetogenic stresses, and/or improve cadaveric islet culture for optimal transplantation results. Several important questions still need to be answered toward these goals (Box 1). It will be exciting to see what new research will reveal in the coming years.

**Box 1.** Open questions in islet architecture

Are determinants of islet architecture conserved between different species? If so, how is species-specific architecture (i.e., rodent *vs*. human) determined?
What are the causes of architectural changes in islets in pregnancy and diabetes, and are islet architectural changes under different physiological conditions adaptive or pathological?
Does architecture determine islet cell type composition (ratios of different endocrine cell types), or does cell type composition determine the architecture?
Are different β cell sub-populations such as, for example, hub/leader cells,^162^ mature/immature β cells,^112,163,164^ extreme β cells,^15^ and other β cell populations dependent on architecture? If so, will achieving correct architecture establish correct β cell subpopulations in stem cell-derived islet-like clusters *in vitro*?
